# Relationship of depression, disability, and family caregiver attitudes to the quality of life of Kuwaiti persons with multiple sclerosis: a controlled study

**DOI:** 10.1186/1471-2377-7-31

**Published:** 2007-09-18

**Authors:** Asmahan F Alshubaili, Abdel W Awadalla, Jude U Ohaeri, Asser A Mabrouk

**Affiliations:** 1 Department of Neurology, Ibn Sina Hospital, PO Box 2547, Safat, 13115, Kuwait; 2 Department of Psychiatry, Faculty of Medicine, Kuwait University, PO Box 24923, Safat, 13110, Kuwait; 3 Department of Psychiatry, Psychological Medicine Hospital, Gamal Abdul Naser Road, PO Box 4081, Safat, 13041, Kuwait

## Abstract

**Background:**

Assessment of subjective quality of life (QOL) of persons with multiple sclerosis (MS) could facilitate the detection of psychosocial aspects of disease that may otherwise go unrecognized. The objectives of the study were to (i) compare the QOL ratings of relapsing remitting (RRMS) and progressive (PMS) types of MS with those of a general population group and the impression of their family caregivers; and (ii) assess the association of demographic, clinical, treatment, depression, and caregiver variables with patients' QOL.

**Methods:**

Consecutive clinic attendees at the national neurology hospital were assessed with the 26 -item WHOQOL Instrument, Beck's Depression Inventory and Expanded Disability Scale. Caregivers rated their impression of patients' QOL and attitudes to patients' illness.

**Results:**

The 170 patients (60 m, 109 f) consisted of 145(85.3%) with RRMS and 25 with PMS, aged 32.4(SD 8.8), age at onset 27.1(7.7), EDSS score 2.9 (1.8), and 76% were employed. The patients were predominantly dissatisfied with their life circumstances. The RRMS group had higher QOL domain scores (P < 0.001), and lower depression(P > 0.05) and disability (P < 0.0001) scores than the PMS group. Patients had significantly lower QOL scores than the control group (P < 0.001). Caregiver impression was significantly correlated with patients' ratings. Depression was the commonest significant covariate of QOL domains. When we controlled for depression and disability scores, differences between the two MS groups became significant for only one (out of 6) QOL domains. Patients who were younger, better educated, employed, felt less sick and with lesser side effects, had higher QOL. The predictors of patients' overall QOL were disability score, caregiver impression of patients' QOL, and caregiver fear of having MS.

**Conclusion:**

Our data indicate that MS patients in stable condition and with social support can hope to have better QOL, if clinicians pay attention to depression, disability, the impact of side effects of treatment and family caregiver anxieties about the illness. The findings call for a regular program of psychosocial intervention in the clinical setting, to address these issues and provide caregiver education and supports, in order to enhance the quality of care.

## Background

Multiple sclerosis (MS) is a chronic debilitating disease, arising from inflammation and demyelination of nerves in the central nervous system, characterized by remissions and relapses, affecting mostly young people and resulting in various degrees of physical and social disability. Although there is currently no cure for the disease, the available immuno-modulatory treatments may slow the appearance of new symptoms[1,2].

In recent times, attention has been focused on health-related quality of life (QOL) as an outcome measure in MS because the concept assesses the broader impact of MS and might indicate less obvious disease burdens[1,3,4]. In this regard, some of the areas of concern in the literature include the impact on QOL, of depression, physical disability [5-8], disease progression [3], neuropathic pain [9], drug side effects [1,2], as well as sexual, bladder and bowel dysfunctions[10]. Other issues of concern are the impact of social support, unawareness of illness [11], cognitive impairment [12], caregiver attitudes/burden[5,13,14], and financial costs [15-17].

With regard to the above issues, the predominant findings are that, compared with the general population, QOL deteriorates early in the course of MS [18-20], and those with the relapsing remitting type have higher QOL than the progressive types[21,22]. In addition, the most important predictors of QOL are depression, physical disability and disease progression[3,5-7,23]. The contribution of socio-demographic factors[8,24] and drug side effects [1,2] is controversial, and financial burden increased with disease progression [15-17].

A review of the literature showed that, with the exception of the report from Iran [6], all the reports on QOL in MS have emanated from the temperate/Mediterranean cultures of Europe and North America, where the disease is traditionally thought to have a higher prevalence and severity[25], compared with countries in the lower latitudes, such as the Arab world[26,27]. Kuwait, a city-state in the Persian Gulf, is one of the relatively low latitude countries where a rising incidence and prevalence of MS has recently been reported[26]. For example, while the prevalence and incidence of MS in Europe are estimated to be 83 per 100,000 and 4.3 cases per 100,000, respectively[25], the figures for the total population in Kuwait are 14.77 per 1000,000 and 2.62 cases per 100,000, respectively[26] (increased from pre-1993 levels of 6.68 and 1.05, respectively, for prevalence and incidence). Specifically for Kuwaiti nationals, the mean incidence rate between 1993 and 2000 was 3.45 per 100,000 population per year (for women it was 7.79/100,000), and the prevalence rate for the same period was 31.15 per 100,000 population [26].

A study of QOL among Kuwaitis is valuable because it presents the perspectives from a country where (compared with the western world) the disease seems to have an earlier age at onset, is thought to have relatively milder clinical severity [26,27], an effective national social welfare system is in place, and family social support is much available in the conservative culture. As a result of the national social welfare system, the Kuwaiti patients have had over a decade experience of free availability of the immuno-modulatory drugs. It would, therefore, be interesting to see whether all these favorable factors would make for good QOL among the MS patients, in comparison with a socio-demographically matched general population sample.

Our review of the literature showed that there is paucity of information on the relationship of family caregiver's attitudes to the illness and caregiver's impressions of the patient's QOL, with the QOL of the patient [5,13]. This perspective is important because the psychological literature on "expressed emotions" (i.e., the impact of emotional interactions in the family on clinical outcome) has consistently shown that family caregiver's emotional appraisal of the patient has an impact on clinical outcome [28]. Katschnig [29] has suggested that it is necessary to involve family members for additional views on aspects of QOL. This issue is particularly important in MS because of the widely reported cognitive impairment among the patients[12], the consequent unawareness of functional deficit, and their impact on the well-being of the patient and family caregiver [13].

The objectives of our study were as follows:

- Using the 26-item WHO QOL Instrument (WHOQOL-Bref), to compare the QOL ratings of relapsing remitting (RRMS) and primary/secondary progressive MS (PMS), with those of a socio-demographically matched general population sample and the impression of their family caregivers (FC);

- to assess the association of the following variables with the patients' QOL: demographic factors, type of drug treatment, side effects of treatment, duration of illness, depression, physical disability, and caregiver attitudes to the patient's illness;

- to examine the concordance between the ratings of the patients and the family caregivers' impressions of the patients' QOL;

- to assess the characteristics of the patient and illness, as well as family caregiver impressions and attitudes that can predict the patient's subjective QOL.

Based on evidence from the literature, we hypothesized as follows: First, in view of available family and national social supports, most patients would be satisfied with QOL items related to family supports and the affluent national economy, but not with items related to health and general well being. Patients with RRMS would have significantly higher QOL domain scores than those with PMS, while all MS patients would have significantly lower scores than the matched general population control group. Second, socio-demographic variables and type of drug treatment would have no significant association with QOL. However, patients with shorter duration of illness, who had no side effects of treatment, scored low on the depression and disability scales, and whose family caregivers had positive attitudes to the illness, would have higher QOL scores[30].

Third, there would be highly significant concordance between patient's ratings and caregiver's ratings of the patient's QOL. Fourth, the most significant predictor of the patient's QOL would be the caregiver's impression of the patient's QOL [31-34].

The clinical relevance of these hypotheses is that they could help to define a subset of stable patients whom clinicians need to give focused attention, and identify the characteristics of patients which psychosocial intervention should target to make for improved quality of care.

## Methods

### Operational definitions

We accepted the WHO definition of QOL as individuals' perception of life in the context of the culture and value system in which they live and in relation to their goals, expectations, standards and concerns[35]. Our focus was on subjective QOL, as distinct from objective QOL[36]. We defined subject's satisfaction as the level of positive appreciation for each item. We quantified each group's satisfaction with each item as at least 50% of respondents in the group positively appreciating the item (i.e., proportion of subjects in the group rating satisfaction for the item as "satisfied" or "very satisfied") ; dissatisfaction (< 50%); bare satisfaction (50 – 65%); moderate satisfaction (66 – 74%); and highest satisfaction (≥ 75%)[31,36].

#### The setting

The study took place at the outpatient clinic of the Neurology Department, Ibn Sina Hospital, which is the national hospital for neurology and neurosurgery in Kuwait. This hospital provided the sample for the epidemiologic studies of MS[26,27], and so we can examine how our clinic sample is representative of the total population of MS patients in Kuwait. All procedures and treatments administered to Kuwaiti nationals are free-of-charge, including hospital registration. The hospital is highly equipped with radiologic (e.g., MRI), neurophysiologic (e.g., evoked potentials), and laboratory facilities, as well as consultant neurology staff and immuno-modulatory drugs.

#### Subjects

The patients were consecutive outpatient clinic attendees who fulfilled the study's inclusion criteria. First, the patients had been formally diagnosed for at least six months, using the Poser Criteria [37], and were now attending the clinic for routine follow-up or exacerbation of illness. However, patients with a flare-up of illness were hospitalized and interviewed only when they had become stable. That is, all patients were in stable clinical condition at the time of interview. Second, each patient was accompanied by at least one family member or friend who lived with them, was responsible for caring for them at home and could complete the questionnaires in Arabic.

The general population group was selected by quota sampling from our WHOQOL-Bref data base for Kuwait, to match the patients by gender, age, occupation, marital status and level of education.

### The WHOQOL – Bref

This is a 26 -item self -administered generic questionnaire, a short version of the WHOQOL – 100 scale[35]. It emphasizes subjective experiences (i.e., subjective QOL) rather than objective life conditions (or objective QOL)[36]. It was developed in a wide range of cultural and clinical settings, including neurology. It is made up of domains and facets (or sub – domains). Domains are broad groupings (e.g., physical/psychological) of related facets. The items on "overall rating of QOL" (OQOL) and subjective satisfaction with health, are not included in the domains, but are used to constitute the "general facet on health and OQOL". There are two models of the WHOQOL -Bref. One model has six domains, namely, physical health, psychological health, level of independence, social relationships, environment, and spiritual. To derive the second (4 – domain) model, the domain of level of independence was merged with that of physical health, while the "spiritual" domain was added to the psychological.

### Modification of the WHOQOL – Bref for the impression of caregivers

In order to produce the version of the WHOQOL – Bref with which the family caregivers rated their impression of the patients' QOL, we used the method of Sainfort's group[38], by giving a new direction to each item, so that the caregiver could rate the patient as an observer. The modification of the WHOQOL – Bref was thus minimal[31].

The internal consistency of the WHOQOL -Bref, as assessed by Cronbach's alpha coefficient for the responses of all subjects, was very high for the patients (0.94), the caregivers' impressions (0.93), and the general population control group (0.90).

#### Other assessments

The patients were also assessed with the 21 -item Beck's Depression Inventory (BDI) [39] and the expanded disability status scale [40]. In addition, we assessed the presence of the following side effects of treatment : influenza -like feeling after injections (e.g., fever, muscle and joint aches); inflammation at site of injection (redness, pain and heat at site of injection); sadness and suicidal ideation; tiredness and fatigue; difficulty in breathing and palpitations; diarrhoea, constipation and nausea. The response options for this assessment of side effects were: never; rarely; sometimes; most times; always. On the basis of clinical experience with the relatives, we assessed their attitudes to the patients' illness by seeking their responses to the following items: caregiver feeling sad about the patient's illness; caregiver feeling disgusted about patient's illness; caregiver feeling tired and exhausted about caring for the patient; and caregiver feeling anxious about the possibility of having MS. The response options were: not present; a little; moderately; a lot.

The internal consistency of the BDI and caregiver attitudes' questionnaire, as assessed by Cronbach's alpha coefficient for the responses of all subjects was high: 0.93 for the BDI and 0.74 for the caregiver attitudes' questionnaire.

#### Data collection procedure

The questionnaires were translated into Arabic by the method of back – translation and have been used in recent studies in an Arab country to assess psychiatric and diabetes patients and their family caregivers [31-34]. In a pilot exercise, the instruments were found to be suitable to the cultural setting.

The WHOQOL questionnaires, the BDI, side effects and attitudes of caregivers' questionnaires were administered by a female native Arab research assistant. One neurologist made all the EDSS assessments. At the preliminary stage of the study, the research assistant was trained in the use of the questionnaires using patients who did not participate in the main study. The study commenced when the research team was satisfied that the research assistant could confidently administer the questionnaires to patients. Patients and caregivers completed the questionnaires privately and without interference from the research assistant, after clarification of the objectives of the study and the meaning of the items. Illiterate patients were assisted by their educated relatives to complete the questionnaire, after the caregiver had completed his or her own. Literacy in Arabic language is very high in Kuwait.

The study was carried out in compliance with the Helsinki Declaration. Ethical approval for the work was obtained from the Faculty of Medicine, Kuwait University, and Ibn Sina Hospital, Kuwait. Patients and family caregivers gave verbal informed consent after the objectives of the study had been explained to them. They were duly informed that there would be no negative consequences for declining to participate. As is well known in our culture for such non-invasive studies[31], all families approached freely consented to participate in the study, especially as the approach was made by clinic staff in charge of the cases.

The physician in-charge of each case assisted the research assistant to record the relevant clinical data. The type of drug treatment was recorded.

#### Data analysis

Data were analyzed by the SPSS – version 11. For the first hypothesis, the pattern of frequency counts was used to assess group satisfaction with QOL items. Summary scores were generated by organizing the items of the WHOQOL-Bref into the six domains and four domains previously highlighted. We compared mean differences in domain scores for the relapsing remitting and the progressive MS types by independent sample t-test. A similar analysis was done with the total scores of the BDI and the EDSS score. QOL domain scores of the patients (as a group) were compared with those of the matched general population group using independent sample t-test. For the second hypothesis, the relationship between age, duration of illness, depression, EDSS score and QOL domain scores was assessed by Pearson's correlation. The association between other socio-demographic variables (level of education, occupation and marital status), type of drug treatment and QOL was assessed by one-way ANOVA. In view of the fact that a number of the socio-demographic and clinical variables were significantly associated with QOL domain scores in these uni-variable analyses, analysis of covariance (ANCOVA) was used to control for the impact of these variables on the differences in QOL domain scores between the two MS groups. For the third hypothesis, the concordance between patient's WHOQOL-Bref ratings and the impression of the caregiver was assessed in two ways. First, we used Kendall's tau to examine the correlation between the corresponding QOL items and domain scores (Kendall's tau was preferred because it is more conservative than Pearson's correlation, as it takes ties into consideration). Second, we used intra-class correlation to examine the internal consistency of the patient -caregiver rating. For the fourth hypothesis, the predictors of patients' QOL (based on patients' general facet on health & QOL as dependent variable) was assessed in step-wise regression analysis. Missing data were handled by excluding cases analysis by analysis. Effect size calculations were used for verification in the cases where it was suspected that a test of significance could have led to a statistical error because of sample size. All tests were two-tailed. A Bonferroni correction (P = 0.01) was used for multiple tests; otherwise, the level of statistical significance was set at P < 0.05.

## Results

### Socio-demographic and clinical characteristics (Tables 1 &2)

**Table 1:** Gender differences in patients' socio-demographic characteristics versus general population control group

Variables	Men (% or SD) N = 60 (1)	Women (% or SD) N = 109 (2)	X^2^	Df	P	Control: men N = 61(%/SD) (3)	Control: women N = 110(%/SD) (4)	Gender differences b/w pts & control P level
Education: N = 60/109			1.30	2	0.52			
Primary/high school	35(58.3)	54(49.6)				38(62.3)	47(42.7)	1 Vs 3: P < 0.08
College/PG	25(41.7)	55(50.5)				23(37.7)	63(57.3)	2 Vs 4: P < 0.4
Occupation: N = 50/83			5.70	3	0.13			
Unemployed/student	14(28.0)	37(44.6)				17(27.9)	54(49.1)	1 Vs 3: P < 0.9
Medium/high skill	36(72.0)	46(55.5)				45(71.6)	56(50.8)	2 Vs 4: P < 0.6
Marital: N = 60/108			0.35	2	0.84			
Single	32(53.3)	58(53.7)				25(40.9)	52(47.2)	1 Vs 3: P < 0.2
Married	28(46.7)	50(46.3)				36(59.0)	58(52.7)	2 Vs 4: P < 0.4
Age: N = 58/108	32.4(7.6)	32.5(9.4)			ns	32.3(7.6)	32.7(9.1)	ns
Age onset of illness	26.9(6.8)	27.3(8.2)			ns			
Duration illness(yrs): N = 60/109	5.7(5.4)	5.2(5.3)			ns			
Disability score* (EDSS): N = 60/108	3.2(2.1)	2.4(1.6)	2.7	166	0.008			
Depression score: (BDI): N = 49/97	14.0(11.4)	14.4(11.2)			Ns			

* EDSS classification (N = 169): Mild: 1–3.5 (139 or 82.2%); moderate: 4 – 6.5 (22 or 13.0%); severe: 7–9 (8 or 4.7%) Mean EDSS = 2.7 (1.8); median = 2.5; mode = 1.0

**Table 2:** Diagnostic differences in socio-demographic characteristics

Variables	Relapsing remitting (% or SD)	Progressive types (% or DS)	X^2^or T	Df value	P value
Education: N = 145/25			2.9	2	ns
Primary/high school	67 (46.2)	16 (64.0)			
College/PG	73 (50.3)	8 (32.0)			
Occup: N = 114/20			28.1	3	0.001
Unemployed/student	23 (20.2)	9 (45.0)			
Medium/high skill	57 (50.0)	1 (5.0)			
Marital: N = 144/25			0.23	2	ns
Single	64 (44.4)	10 (40.0)			
Married	66 (45.8)	12 (48.0)			
Gender: N = 60/109			8.9	1	0.003
Men	44 (73.3)	16 (26.7)			
Women	100 (91.7)	9 (8.3)			
Age: N = 144/23	31.9 (8.8)	35.9 (7.5)	2.1	165	0.04
Age onset illness	27.3 (7.9)	26.2 (6.3)	0.6		ns
Duration illness (yrs)	4.6 (4.2)	9.8 (8.1)	4.8	168	0.001
Disability: N = 144/25	2.2 (1.1)	5.9 (1.9)	13.8	167	0.001
Depression: N = 126/21	13.7 (10.6)	16.7 (14.4)	1.1		ns*

* Effect size = 0.27 (95% C.I. = -0.20 – 0.73)

Over a period of seven months, 170 consecutive attendees at the clinic met our inclusion criteria and agreed to participate in the study. They consisted of 60 men (35.5%) and 109 women (64.5%) (the gender of one patient was not recorded). Most (145 or 85.3%) had relapsing remitting MS (RRMS), while 22 (12.9%) and 3 (1.8%) had secondary progressive and primary progressive MS (PMS), respectively. Of those with RRMS, 100 (69.4%) were women, while women constituted 9(36%) of those with SPMS. They were aged 32.4 (SD 8.8) years (range 16 – 55, median 31). Age at onset of illness was 27.1 (SD 7.7, median 26.0) years. Duration of illness was 5.4 (SD5.3) years (range six months to 30 yrs, median 4 yrs). They had been on treatment for 2.9 (SD2.6) years (range six months to 14 yrs, median 2.5 yrs). The mean EDSS score was 2.7 (SD 1.8, median 2.5, mode 1.0). Using EDSS scores, 139 (82.2%) had mild disease (EDSS score 1–3.5), 22 (13.0%) had moderately severe disease (4 – 6.5), and 8 (4.7%) had severe disease (7–9). While the men had significantly higher EDSS scores (P = 0.008), there were no significant gender differences in age, education, occupation, marital status, age at onset of illness, duration of illness and depression (BDI) score (P > 0.05). The rate of formal unemployment was 32 (23.9%). Table 1 shows that the patients were well matched with the general population control group (N = 171), by gender, age, education, occupation and marital status (P > 0.05).

Table 2 shows that those with RRMS tended to be younger (P = 0.04), in formal employment (P < 0.0001), had been ill for a shorter period (P < 0.0001), and had much lower disability scores (P < 0.0001).

### Satisfaction and QOL domain scores (Tables 3 &4)

**Table 3:** Comparative level of group satisfaction with QOL items

		3A: Patients	
Highest satisfaction (≥ 75% subjects)	Moderate satisfaction (66 – 74% subjects)	Bare satisfaction (50–65% subjects)	Dissatisfied (< 50% of subjects)
Satisfaction with transport (76%).	Satisfaction with money to meet needs (66%).	Health satisfaction(57), life meaningful(56) Safety(57), health environ(58), bodily appearance (53), sleep(52), ADL(53), self-satisfaction(58), personal relations(55), friends' support(50), living place satisfaction(63), access to health service(55).	OQOL(41), feeling pain(47), medical treatment need(25), enjoy life(46), ability to concentrate(39), energy(33), information for health(42), leisure activities(45), ability to get around(46), work capacity(46), sex satisfaction(45), negative feelings(17).
		3B: General population control group	
Highest satisfaction (≥ 75% subjects)	Moderate satisfaction (66 – 74% subjects)	Bare satisfaction (50–65% subjects)	Dissatisfied (< 50% of subjects)
OQOL(81%), ability to get around(82%), work capacity(77%)	Health satisfaction(73%) medical treatment need(69), self-satisfaction(74), personal relations(70), sex satisfaction(66), living place satisfaction(72)	Feeling pain(51), life meaningful(60), feeling safe(60), energy for life(55), sleep(58), ADL(65), support from friends(50)	Enjoy life(44), ability to concentrate (40), healthy environment around(49), money(42), information (39), leisure activities(26), access to health service(45), negative feelings (17%).

Note: OQOL = overall QOL; ADL = activities of daily living

**Table 4:** Comparison of QOL domains by gender and MS type versus matched control groups

A. By gender:						
WHOQOL-Bref domains	MS men(SD): 1	MS women(SD): 2	P level	Control men(SD): 3 N = 61	Control women(SD): 4 N = 110	P level differences b/w pts & control groups: 1 vs 3; 2 vs 4, respectively*
Physical health: N= 58/108	9.8(2.6)	9.6(2.3)	Ns	11.2(1.8)	10.5(2.0)	P < 0.0008; 0.002
Psychol health: N= 59/109	15.6(3.5)	15.9(3.8)	Ns	18.1(2.4)	17.1(2.9)	P < 0.0001; 0.009
Independence: N= 57/109	12.9(3.7)	12.6(2.9)	Ns	16.1(2.4)	15.7(2.4)	P < 0.0001; 0.0001
Social relations: N= 58/105	9.6(2.8)	9.8(2.6)	Ns	11.5(1.7)	10.8(2.5)	P < 0.0001; 0.006
Environment: N= 57/102	27.5(5.4)	27.5(5.1)	Ns	28.3(4.4)	28.3(5.3)	P = 0.4; 0.3
Spiritual N = 59/108	3.2(1.0)	3.5(1.0)	Ns	3.7(0.9)	3.7(1.0)	P < 0.005; 0.2
General facet: N= 59/107	6.9(1.6)	6.7(1.7)	Ns	8.1(1.2)	7.9(1.5)	P < 0.0001; 0.0001
4- domain physical health	22.6(5.7)	22.1(4.8)	Ns	27.3(3.8)	26.3(3.9)	P < 0.0001; 0.0001
4- domain psychol health	18.8(4.3)	19.3(4.5)	Ns	21.8(3.1)	20.8(3.8)	P < 0.0001; 0.009

B. By MS type						
WHOQOL-Bref domains	RRMS (SD): 1	PMS (SD): 2	T	P level	Control (SD): 3 N = 171	P level differences b/w pts & control groups: 1 vs 3; 2 vs 3, respectively**

Physical health: N = 141/25	10.0(2.1)	7.6(2.9)	5.1	0.001	10.8(1.9)	P < 0.005; 0.0001
Psychol health: N = 143/25	16.4(3.1)	12.0(4.8)	5.9	0.001	17.4(2.8)	P < 0.003; 0.0001
Independence: N = 142/24	13.2(2.7)	9.2(4.1)	6.3	0.001	15.9(2.3)	P < 0.0001; 0.0001
Social relations: N= 140/24	10.3(2.2)	6.5(3.2)	7.2	0.001	11.1(2.2)	P < 0.002; 0.0001
Environment: N = 135/24	28.4(4.6)	22.4(5.7)	5.7	0.001	28.3(4.9)	P = 0.9; 0.0001
General facet: N = 142/25	7.1(1.2)	5.0(2.3)	6.7	0.001	8.0(1.4)	P < 0.0001; 0.0001

* For significant differences: t ranged from 2.6 to 8.6, df = 117, 215, respectively

** For significant differences: t ranged from 2.9 to 9.5, df = 309, 193, respectively.

Table 3 shows that, using the operational definitions earlier highlighted, the patients were predominantly either dissatisfied or barely satisfied with various aspects of their life circumstances. However, the areas that they were highly satisfied with concerned transportation and availability of money for needs. Accordingly, Table 4 shows that when compared with the general population group by gender and MS type, the patients had much significantly lower QOL domain scores (P mostly < 0.001). However, it is noteworthy that in the case of QOL environment domain (which includes items on money, transport, information), patients had similar scores with the control group by gender (Table 4A), and those with RRMS also had similar scores with the control group (Table 4B) for this domain (P > 0.05). Furthermore, while there were no significant gender differences in QOL domain scores (P > 0.05; Table 4A), those with RRMS had much significantly higher scores than patients with PMS in all the domains (P < 0.0001), using uncorrected scores (Table 4B).

### Factors associated with WHOQOL domain scores in univariate analysis

#### Age

The correlation (Pearson's) between age and QOL domain scores was negative for all the domains, but reached significance for only the following: independence (r = -0.23, N = 141, P = 0.006), and social relations (r = -0.21, N = 139, P = 0.01).

#### Education

Subjects with at least college education had the highest scores, while the illiterate had the least scores in all domains. After Bonferoni correction, this tendency reached significance for only the independence domain (F = 5.9, df = 2/163, P = 0.003).

#### Occupation

Students and those in medium/high skill work scored significantly higher than the unemployed, in the social relations domain (F = 7.0, df = 3/124, P = 0.001), and the general facet on health and QOL (F = 7.0, df = 3/127, P = 0.001).

#### Marital status

There were no significant differences in QOL domain scores for marital status groups (P > 0.05).

#### Duration of illness and treatment

In all the domains, duration of illness and treatment were negatively correlated with QOL domain scores. In the case of duration of illness, the correlation was highly significant for all domains (r ranged from -0.26 to -0.37, P mostly < 0.001), except the spiritual domain (P = 0.04). For duration of treatment the correlation reached significance after Bonferroni correction, for social relations (r = -0.35, P < 0.0001).

#### Disability (EDSS) and depression (BDI)

The correlation between QOL domain scores, on the one hand, and EDSS and BDI scores, on the other hand, was negative. For EDSS, the correlation was highly significant for all domains (r ranged from -0.35 to -0.54, P < 0.0001). The correlations were also highly significant for BDI (r ranged from -0.24 to -0.36), except for the general facet (r = -0.17, P = 0.04).

#### Caregiver attitudes to patient's illness

Patients had a tendency to have lower QOL domain scores and higher depression scores, if the family caregiver had either of the following attitudes: feeling sad about patient's illness, feeling disgusted about the illness, feeling exhausted about caring for the patient and feeling afraid about having the illness. For caregiver feelings of sadness and exhaustion, this tendency did not reach significance (P > 0.05). But it reached significance in the psychological domain, for caregiver fear of illness (t = 2.4, df = 130, P = 0.02), and in the independence domain, for caregiver feeling disgusted about the illness (t = 2.7, df = 124, P = 0.009).

### Association of side effects of treatment with QOL domain scores

#### Influenza -like effects, inflammation and diarrhoea

The tendency for patients with influenza -like effects, inflammation at site of injection and diarrhoea, to have lower QOL scores and higher depression scores, did not reach significance (P > 0.05).

#### Suicidal ideation

Patients who had this complaint " most times" had a tendency to have the lowest QOL domain scores. But this reached significance for only the spiritual domain (F = 6.1 df = 2/147, P = 0.003).

#### Feeling of fatigue

The tendency for patients with this complaint " most times" to have lower QOL reached significance for only the spiritual domain (F = 5.3, df = 2/149, P = 0.006).

#### Difficulty in breathing as side effect of treatment

There was a tendency for those who had this complaint " most times" to have lower QOL scores. This reached significance for the spiritual domain (F = 7.2 df = 2/149 P = 0.001), and general facet (F = 4.2 df = 2/150, P = 0.02)

### Factors associated with QOL domain scores in multivariate relationship (Table 5)

**Table 5:** Factors associated with QOL: significant covariates of QOL domains in ANCOVA*

Significant factors or covariates	QOL domains significantly impacted by covariates	F	P level
Depression: total BDI scores	Psychological health	13.4	0.0001
	Physical health	6.3	0.01
	Independence	10.0	0.002
	Social relations	4.7	0.03
	Spiritual	19.2	0.001
Disability score: EDSS	Physical health	10.3	0.002
	Social relations	4.4	0.04
	Environment	4.1	0.05
Age of patient	Independence	3.9	0.05
	Social relations	5.2	0.02
Diagnosis effect	Social relations	4.2	0.04
	Spiritual	4.1	0.05
	General facet health & QOL	7.6	0.007

* Note: After controlling for these covariates, the large differences in QOL domain scores between the RRMS and PMS earlier highlighted (Table 4), was significant only for the general facet on health & QOL (P < 0.005).

In view of the many univariate relationships, the data were subjected to analysis of covariance (ANCOVA), to examine the factors that impact on QOL in multivariate relationship, and control for the effect of these relationships in the differences in QOL between the RRMS and PMS. Table 5 shows that, of all the above variables, the most important factors associated with QOL domain scores were depression and disability, with age playing a relatively minor role. Of note, the diagnosis effect (i.e., whether patient had RRMS or PMS) was significant mainly for general facet on health and QOL (P = 0.007). Accordingly, when the impact of the variables were controlled for in ANCOVA, to assess QOL domain differences between RRMS and PMS, we found that, the highly significant differences noted in Table 4 became significant only for the general facet (P = 0.005).

### Patient's current feeling of well-being (Table 6)

**Table 6:** Comparison of QOL domains: patients feeling currently well versus feeling currently ill*

QOL domains	Feel currently ill Mean(SD)	Not feel ill currently Mean(SD)	T	Df	P
Physical health: N = 124/40	9.3(2.4)	10.8(2.3)	3.3	162	0.001
Psychological health: N = 125/41	15.3(3.9)	17.1(2.7)	2.7	164	0.007
Independence: N = 124/40	12.2(3.2)	14.1(3.2)	3.3	162	0.001
Social relations: N = 120/42	9.4(2.8)	10.8(2.1)	3.1	160	0.003
Environment: N = 118/40	26.9(5.3)	29.4(4.7)	2.7	156	0.008
Spiritual: N = 124/41	3.2(1.1)	3.9(0.8)	3.6	163	0.001
General facet health & QOL: N = 125/40	6.6(1.7)	7.5(1.3)	3.2	163	0.002
4 -d Physical health: N = 123/39	21.5(4.9)	24.7(5.1)	3.4	160	0.001
4 -d Psychological health: N = 124/41	18.5(4.7)	20.9(3.1)	3.2	163	0.002

* Caregiver impression ratings: The caregivers rated the patients in the same direction, such that, in all domains, patients who felt well were judged to have significantly higher QOL scores. Except the spiritual domain (t = 1.9, P = 0.053), t ranged from 2.4 to 4.4,, df ranged from 138 – 143, P ranged from 0.02 to 0.000 (mostly P < 0.0001).

In all the domains, patients who felt currently well had significantly higher QOL scores (P < 0.01).

### Association of immuno-modulatory treatment with QOL (Table 7)

**Table 7:** QOL domain scores: patients on immuno-modulatory drugs vs patients not on immuno-modulatory drugs

QOL domains	Rebif: 1 N = 14 Mean(SD)	Avonex: 2 N = 75 Mean(SD)	Betaseron: 3 N = 54 Mean(SD)	Other drugs: 4 N = 9 Mean(SD)	F	Df	P	Significantly different groups
Physical health	10.7(2.2)	9.5(2.4)	9.6(2.2)	7.7(2.8	3.1	3/144	0.03	1 > 4
Psychol health	16.3(2.8)	15.5(3.6)	15.6(3.8)	15.4(4.9)			0.9	-
Independence	13.1(2.7)	12.5(2.9)	12.7(3.5)	9.9(3.9)	2.2	3/146	0.05	1 & 3 > 4
Social relations	9.1(2.7)	9.8(2.4)	9.6(2.9)	8.9(2.8)			0.6	-
Environment	26.2(4.2)	27.6(5.3)	27.1(5.0)	25.0(5.4)			0.5	-
Spiritual	3.1(1.2)	3.4(0.9)	3.3(0.9)	3.3(1.4)			0.8	
General facet	7.3(1.5)	6.8(1.6)	6.7(1.9)	5.8(1.9)	2.1	3/147	0.05	1 > 4
Depression(BDI)*	16.3(8.3)	15.4(10.3)	12.3(9.3)	21.6(25.8)	2.0	3/127	0.05	4 > 3*
Disability(EDSS)	3.4(1.3)	2.7(1.7)	2.6(2.0)	4.4(2.5)	2.9	3/147	0.04	4 > 2 & 3

* Effect size (ES) calculations(95% C.I.) : 4 Vs 1: ES = 0.31(- 0.54 – 1.14); 4 Vs 2: ES = 0.49 (- 0.21 – 1.18); 4 Vs 3: ES = 0.73(0.00 – 1.18)

Duration of drug treatment: Mean = 2.9(2.6), range = 0.5 – 14 yrs, median = 2.5 yrs, mode = six months

Only one subject was on glatiramer acetate (copaxone), not included in this analysis.

Those on the three types of immuno-modulatory drugs had similar QOL scores, and their scores tended to be higher than those not on immuno-modulatory treatment. Those on Rebif had significantly higher scores than those not on immuno-modulatory drugs, for physical health, independence and general facet (P < 0.05). A similar trend was noted for depression and disability, where those on immuno-modulatory treatment had lower scores (P < 0.05).

### Concordance of patient's and caregiver's QOL ratings (Tables 8 &9)

**Table 8:** Correlation of patient's rating and caregiver impression for QOL items (Kendal's tau)*

WHOQOL-Bref items	Kt	N	P
Overall rating QOL	0.33	145	0.001
Health satisfaction	0.27	146	0.001
Pain feelings	0.15	144	0.03
Medical treatment need	-0.15	141	0.03
Enjoyment of life	-0.08	144	0.26
Life meaningful	0.19	144	0.007
Ability to concentrate	0.28	144	0.001
Safety in daily life	0.35	144	0.001
Environmental health	0.24	144	0.001
Energy for life	0.36	143	0.001
Bodily appearance	0.33	144	0.001
Enough money for needs	0.31	141	0.001
Available information for health	0.23	142	0.001
Leisure opportunity	0.26	142	0.001
Ability to get around	0.45	142	0.001
Sleep satisfaction	0.24	145	0.001
Activities of daily living	0.23	145	0.001
Work capacity	0.35	146	0.001
Self -satisfaction	0.27	145	0.001
Personal relationships	0.37	144	0.001
Satisfaction with sex life	0.37	141	0.001
Satisfaction with friends' support	0.27	146	0.001
Condition of place of living	0.25	142	0.001
Access to health service	0.14	146	0.06
Satisfaction with transport	0.28	146	0.001
Negative feelings	0.20	146	0.005
QOL domains	R	N	P
Physical health	0.37	141	0.001
Psychological health	0.33	141	0.001
Independence	0.34	137	0.001
Social relations	0.35	137	0.001
Environment	0.40	132	0.001
Spiritual	0.19	144	0.007
General facet health & QOL	0.29	145	0.001

* Intra-class correlation coefficient (ICC) for Pts WHOQOL-Bref & Caregiver impression: ICC = 0.96 (95% C.I. = 0.95–0.97)

**Table 9:** Comparison of patient's ratings and caregiver impression scores for WHOQOL-Bref domains

QOL domains	Patients: Mean(SD)	Caregiver impression: Mean(SD)	T	Df	P
Physical health	9.9(2.1)	9.8(1.8)			ns
Psychological health	16.4(3.1)	15.9(2.3)			ns
Independence	13.1(2.7)	12.8(2.2)			ns
Social relations	10.3(2.3)	10.0(2.3)			ns
Environment	28.4(4.6)	27.0(4.6)	3.1	114	0.002
Spiritual	3.5(0.9)	3.5(0.9)			ns
General facet health & QOL	7.1(1.2)	6.7(1.5)	3.2	124	0.002

Table 8 shows that there was a high degree of concordance between the patient's and caregiver's QOL ratings, both at the level of items (P mostly < 0.001) or at the macro level of domain scores (P mostly < 0.0001). In addition, the intra-class correlation between the total responses of the patients and those of the caregivers was very high (ICC = 0.96, 95% C.I. = 0.95 – 0.97). Accordingly, the significant differences between the QOL domain scores of the patients and those derived from caregiver impression ratings were noted for only the environment domain and general facet (P = 0.002), where the patients rated themselves as having higher scores than the caregivers rated them (Table 9).

### Predictors of overall QOL (Table 10)

**Table 10:** Predictors of patient's QOL: general facet health & QOL as dependent variable*

Dependent variable	Predictors	Variance (%)	Total (%)	Standard Beta	T	P
Patient's general facet on health & QOL	Disability score	34.0	46.9	-0.46	-5.3	0.001
	Caregiver impression general facet health & QOL	10.1		0.32	3.6	0.001
	Caregiver anxious about having MS	2.8		-0.17	-2.2	0.03

* Variables not in the step-wise regression equation: age of patient, duration of illness, age at onset of illness, caregiver feeling sad about patient's illness, caregiver feeling disgusted with patient's condition, caregiver feeling tired/exhausted about caring for patient, BDI total score, general facet for caregiver.

In multiple regression analysis, with the general facet on health and QOL as dependent variable, and all other factors as independent variables, the only significant predictors of patients' overall QOL were, disability status (P < 0.0001), general facet derived from caregiver impression of patients' QOL (P < 0.0001), and caregivers' fear of having the illness (P = 0.03). These accounted for 46.9% of the variance.

## Discussion

### Limitations and strengths of the study

The limitations of the study are that it was cross-sectional, the patients were selected because they had family support, involving only subjects from one center, and so the subjects may not be representative of the general population of MS patients in Kuwait. However, this hospital is the national center for neurology, where the vast majority of MS patients are referred to, and the socio-demographic and clinical characteristics of our patients were much similar to those of the Kuwait epidemiologic sample [26]. The epidemiologic sample consisted of 336 patients (41.7% men, 58.3% women) recruited over a period of seven years (1993–2000) at the same hospital. Our sample of 170 patients was similar to the epidemiologic sample in terms of gender distribution(35.5% men, 64.5% women), age at onset of disease (27.1 Vs 26.0), diagnostic types of MS (RRMS: 85.3% Vs 78.4%), and type of treatment (0.6% of each sample were on glatiramerate acetate, while others were mostly on beta interferon). Hence our sample size and the characteristics of the patients were representative of the Kuwaiti clinical population and sufficient to test the hypotheses of the study. In our review of several recent MS reports from the western world (i.e., western Europe and north America) we found that their clinic samples were usually averagely aged over 41 years, had a mean age at onset of 29.5 to 34.3 (mostly over 30) years, mean EDSS scores were mostly over 4.0 (range 2.8 to 4.5), patients with mild severity (EDSS score 0–3.5) ranged from 21.3% to 47.9%, those with RRMS ranged from 28.9% to 75.3%, and the proportion in employment ranged from 25% to 40% [8,15,16,19,41-44]. In a review of the epidemiology of MS in Europe, it was noted that the prevalence of mildly severe MS was 45 – 55% [25]. When these reported clinical characteristics of MS in the western world were compared with our sample, we found that our data were in line with the impression of clinicians in Kuwait, that our patients are relatively younger, tend to have an earlier age at onset of disease, tend to have a milder severity of illness, and tend to be more able to sustain their employment (Tables 1 &2) [26,27]. It is against this background of apparent differences in clinical characteristics that our results will be compared with western reports.

The other strengths of the study are that we were able to compare the MS groups with a gender-, age-, education-, occupation-, and marital status- matched general population group, and assessed the relationship of caregiver impression of the patient's QOL and caregiver attitudes to the patient, with the QOL of the patient. This is a rare methodology in MS QOL literature. Furthermore, we have shown that despite skepticism about its use in MS[4], the WHOQOL-Bref performed very well in this study, as it significantly discriminated between known groups (e.g., RRMS versus PMS; MS versus control group; patients feeling currently well versus those feeling sick). Also, it had impressive external criterion validity, as the domain scores were highly significantly correlated with depression and disability.

### Comparison of QOL item ratings and domain scores

In analyzing for the first hypothesis (Table 3), we found that the only items of the WHOQOL-Bref that the patients were moderately or highly satisfied with (i.e., > 66% of subjects responded satisfied/very satisfied) were money for needs and transport. Otherwise, they were predominantly dissatisfied with items related to their health condition, such as pain, reliance on treatment, ability to concentrate and ability to get around.

The ratings of the family caregivers about the patients' QOL were largely similar to those of the patients. This response pattern is an indication of the reliability of the ratings of the patients. For, while they were highly satisfied with items related to their materially affluent national circumstances (availability of money and transport), they were dissatisfied with items related to their general health situation. It shows that in rating subjective QOL, the patients and caregivers made realistic appraisals of their life circumstances. However, the patients were barely satisfied with items of social support that we logically expected them to be highly satisfied with in a conservative culture, such as personal relations and support from friends. This response pattern is comparable to what was obtained from psychiatric and diabetic patients in Sudan, a similarly conservative country, but with much lower national material affluence [31-34]. While the Sudanese psychiatric patients and those with type 1 diabetes were not satisfied with any items up to the moderate level, patients with type 2 diabetes were moderately satisfied with items related to life being meaningful, self -satisfaction, and condition of living place. In cross-national European studies, it was found that the level of satisfaction in certain domains appeared to be associated with the local style of living and culture, while other areas appeared to be more independent of local variations [45].

In line with this high level of dissatisfaction, our MS patients had QOL domain scores that were significantly lower than those of the control group in all the domains, except the environment and spiritual domains (Table 4). There were no significant gender differences, and in the uncorrected scores, those with RRMS had much significantly higher scores than those with the progressive types of MS. These findings are in line with the vast majority of reports from the western world and elsewhere [6,18-22]. However, a USA study found that, while patients had lower scores than the general population in most domains, 77% of the patients were mostly satisfied or delighted with their QOL [30], and the patients had similar scores with the general population in some domains.

In comparing the QOL domain scores of our MS patients with those of psychiatric (N = 300) and diabetic (N = 241) patients similarly assessed in Sudan [31,33], we found that our Kuwaiti patients had similar scores with the Sudanese diabetic patients for the following domains: general facet (mean 6.8 in each case), physical health (mean 9.7 in each case), and independence (mean 12.6 in each case). Also, the Kuwaiti MS patients had similar scores with the Sudanese psychiatric patients for the following domains: social relations, spiritual, psychological health and physical health (P > 0.05). However, the Sudanese diabetic patients had significantly higher scores than the Kuwaiti MS patients for the following domains: social relations (P < 0.04), spiritual (P < 0.009), and psychological health (P < 0.002). The Kuwaiti patients had significantly higher scores than the Sudanese diabetic (P < 0.03) and psychiatric (P < 0.001) patients for the environment domain. While the higher Kuwaiti score for the environment domain (which includes money and transport) is a reflection of their better material circumstance, the higher Sudanese score for social relations domain was a surprising finding, in view of the presumed higher social support in Kuwait. We shall seek to explain this finding later.

Although the relatively lower QOL domain scores of our patients in comparison with the general population is in line with the literature, and shows the adverse impact of MS on psychosocial functioning even for those with milder disease course, we were still surprised that our patients showed such a high level of dissatisfaction with their life circumstances, as expressed in the items of WHOQOL-Bref. However, in a recent report on posttraumatic stress disorder (PTSD) among Kuwaiti veterans of the first Gulf War and their wives, it was found that, despite a generous national welfare and family social supports, the prevalence of PTSD remained high among the sample, six years after the war [46]. In other words, health impairment can adversely affect a patient's capacity to benefit from available social support, especially as physical health is a strong correlate of subjective well-being [47], and it has been shown that the association between social resources and life satisfaction is mediated through health impairment [48,49]. In a study of social support as a mediator in the relationship between functional status and QOL, it was found that impairment was associated with fewer friendship contacts, fewer family contacts, less perceived belonging and less perceived tangible aid [50].

### Factors associated with QOL

In analyzing for the second hypothesis, we found that depression and disability were the most important factors in multivariate relationship. This finding has been robustly replicated in the literature [3,5-7,19,23,44]. Accordingly, we found that when these two factors were controlled for, the previously noted differences between the RRMS and PMS became significant for only the general facet on health and QOL. The implication of this finding is that the clinician has to routinely assess, treat and counsel the patient for depression and limitation of movements. Some authors [51] have considered that the high level of correlation between BDI and QOL domain scores indicates measurement overlap, and have suggested that the assessment of subjective QOL should always be checked for the influence of depressive symptomatology on QOL scores. Our ANCOVA operation adequately addressed this issue.

In the case of side effects of treatment, the noteworthy finding was that those who were worried by the effects of treatment were significantly more likely to feel that life was not meaningful (i.e., lower score on spiritual domain). On the other hand, those on immuno-modulatory drugs and those who felt currently well consistently tended to have higher QOL domain scores, lower depression scores, and lower disability scores, compared with those who were not on immuno-modulatory treatment (Tables 6 &7). In other words, while the patients should be encouraged to be compliant with treatment, they should be routinely monitored for side effects of treatment.

### Concordance of patient -caregiver ratings of patient's QOL

In analyzing for the third hypothesis, we found that there was a high degree of concordance between the ratings of the patients and family caregivers' impression of the patients' QOL (Tables 8 &9). Coupled with the high reliability indices of the questionnaires, this is an indication that this sample of patients was realistic in their responses and did not show evidence of unawareness of functional deficit [13]. The findings are in line with the reports from Sudan on similarly assessed patients [31-34].

### Predictors of QOL

In analyzing for the fourth hypothesis, we found that the predictors of general facet on health and overall QOL were disability status, family caregiver impression of the patients' QOL, and caregiver being anxious about the possibility of having the illness (Table 10). The predictive power of caregiver impression has now been replicated for patients with chronic medical illnesses in Sudan[31-34,52] and Kuwait, and should therefore be regarded as noteworthy. The implication of this finding is that the clinician should assess and address family caregivers' anxiety over fear of developing the illness, and improve their awareness about the nature and management of multiple sclerosis. We suggest that recent brain -behavior findings about "mirror neurons"[53] and the phenomenon of "social intelligence"[54] indicate that the patient- caregiver dyad interaction and its association with QOL has roots in the neurology of human behaviour [52-54].

## Conclusion

Our data indicate that MS patients in stable clinical condition and with social supports can hope to have better QOL, if clinicians pay attention to depression, disability, the impact of side effects of treatment and family caregiver anxieties about the illness. Patients who are older, less educated and unemployed are particularly vulnerable and need specific attention. The findings constitute an evidence base for the establishment of a regular program of psychosocial intervention in our clinical setting, to address these issues and provide caregiver education and supports, in order to enhance the quality of care.

## Competing interests

The author(s) declare that they have no competing interests.

## Authors' contributions

AFA, AWA and JUO jointly designed the study, analyzed the data and wrote up the manuscript. AAM made the clinical assessments, including EDSS. AWA and JUO trained the research assistant. AFA and AAM supervised the interviews, and ensured correct diagnosis and other clinical data. All authors read and approved the manuscript.

## Pre-publication history

The pre-publication history for this paper can be accessed here:


